# Sensitivity of Line-of-Sight Estimation to Measurement Errors in L-Shaped Antenna Arrays for 3D Localization for In-Orbit Servicing †

**DOI:** 10.3390/s25133946

**Published:** 2025-06-25

**Authors:** Botond Sándor Kirei, Vlad Rațiu, Ovidiu Rațiu

**Affiliations:** 1 Basis of Electronics Department, Technical University of Cluj-Napoca, 400114 Cluj-Napoca, Romania; vlad.ratiu@cs.utcluj.ro; 2 Control Data Systems SRL, 400267 Cluj-Napoca, Romania; ovidiu.ratiu@cds.ro

**Keywords:** in-orbit servicing, inter-satellite link, L-shaped antenna array, sensitivity analysis, 3D localization/positioning

## Abstract

The sensitivity analysis of line-of-sight estimation to measurement errors in the L-shaped antenna array contributes to the deeper understanding of how the measurement errors affect a 3D localization system aimed to be used in the next generation of inter-satellite links. First, the proposed 3D localization model in the Cartesian coordinate system is given, where, for simplicity, the origin of the coordinate system is the origin of the L-shaped antenna array. The proposed localization method relies on three measurements: range measurement and line-of sight angles with the x- and y-axis, respectively. The sensitivity analysis revealed that the variation in the L-shaped antenna array geometry (variation of the antennas placements) has an impact on the 3D positioning precision: a misplaced antenna—placed closer than intended—will have a larger line-of-sight error for small distances/ranges in the presence of range measurement errors. Notably, a misplaced antenna will result in a larger line-of-sight error for large distances/ranges in the presence of phase measurement errors.

## 1. Introduction

Inter-satellite links (ISLs) form the backbone of satellite constellations and are essential for spacecraft rendezvous [1]. While reliable communication between spacecraft is critical, traditional data links are no longer sufficient. Modern ISLs are expected to support additional functionalities, such as localization and positioning measurements, including ranging and angle of arrival (AoA) determinations. An emerging use case of ISLs is given by the in-orbit-servicing (IOS) operations. The main scope of IOS is extending the life cycle and functionalities of spacecrafts that are already in orbit [2] by providing in-orbit maintenance, adjusting a satellite’s/spacecraft’s orbit, changing the direction it is facing, providing in-orbit refueling/recharging, or even changing or upgrading the instruments onboard. Moreover, commercial operators must be involved in future IOS operations, opening the way for new jobs, new business opportunities, and, foremost, commercial sustainability. IOS is enabled by several technologies: mechanical and electrical coupling interfaces (with a potential option on refueling), navigation for automatic rendezvous and docking, and ISLs for cooperative rendezvous and docking. At present, the extensive standardization effort of all these components is carried out with the important issue of the interoperability of the several participants in the IOS scenarios. In the docking/birthing scenarios, the ISL must be “gifted” with important functionalities: low latency data communication, metrology (to support coarse and fine ranging, angle of arrival (AoA), or line-of-sight (LoS) measurement), and safety functionalities for collision avoidance. The prospect of existing space communications standards brings out several recommendations from the Consultative Committee for Space Data Systems (CCSDS). According to CCSDS recommendation [3], two major standards paths are recommended: the Wi-Fi Alliance certifications (heavily drawn from IEEE 802.11 standards [4]) and the 3rd Generation Partnership Project (3GPP)—Long Term Evolution (LTE) and beyond—standards for mobile High Data Rate Wireless Proximity Network Communications architecture, protocols, and communication standards in support of activities associated with space missions. Another relevant and live recommendation from the CCSDS is the Proximity-1 network protocol [5,6,7,8]. The Proximity-1 network protocol has two significant short comings when it comes across IOS: (i) the lack of support for networking: a peer-to-peer communication link between the two spacecraft is a minimum requirement, but the possibility of multiple access networking between a servicer and multiple serviced spacecraft should be foreseen (a servicer could guide multiple serviced vehicles at the same time or a servicer can have several docking stations—like a “gas-station” scenario); (ii) the lack of support for navigational assistance: coarse/fine ranging and LoS are necessary features to assist in the spacecraft’s guidance and with the navigation controller (GNC) before the optical link is established and as a back-up system after the optical link is established; (iii) the lack of support for collision avoidance: although space debris handling is not necessarily related to IOS, it is a constant headache for current space operators looking for cleaning up the increasingly crowded orbits [9]; (iv) the lack of network time distribution that may be used for timestamping events in the local device. Thus, we see a clear technological advantage in developing an ISL system that also provides ranging information and supports the spacecraft with line-of-sight (LoS) angle and relative tilt measurements [10].

Nowadays, ranging is enabled in ISL communication by the deployment of pseudorandom coded modulation [11] with either Gaussian Minimum Shift Keying (GMSK) or Phase Shift Keying (PSK) [12], but these procedures only offer ranging without the capability for AoA measurement.

Traditionally, radio detection and ranging (RADAR) systems are aimed at determining the range—or the distance—and the AoA of a target. Fortunately, there is a myriad of AoA methods [13]; the key methods are as follows: conventional beamforming (utilizes an array of antennas to form a beam pattern, focusing on a specific direction; the AoA is estimated by steering the beam in different directions and identifying the direction with the maximum received signal power); multiple signal classification (MUSIC) uses the eigen structure of the covariance matrix of received signals to identify signal directions [14]; estimation of signal parameters via rotational invariance techniques (ESPRIT) [15]; Capon’s Method [16] is based on an adaptive beamforming technique that enhances resolution by forming a spatial filter, focusing on minimizing interference and noise while maintaining the signal from a particular direction); the matrix pencil method [17] uses a matrix decomposition approach to estimate the AoA by considering the signal as a sum of complex exponentials; Time Difference of Arrival (TDOA) measures the difference in the time that it takes for a signal to reach different sensors; AoA measurements using machine learning [18] techniques like neural networks and support vector machines are employed; finally, phase interferometry [19] measures the phase difference of received signals at different antennas to estimate the AoA.

Simple AoA is most likely to act just on a plane, giving information about the direction of the source/target, without the elevation/azimuth of the source. Three-dimensional; antenna arrays, such as an L-shaped antenna array [20], can offer both direction and elevation information. As will be shown in later sections, a hybrid-ISL (H-ISL) system was proposed that can perform (i) precision range measurements based on the combination of a course and fine range estimation and (ii) LoS estimation based on the simplest L-shaped antenna array. Furthermore, the sensitivity of LoS estimation to measurement errors in the H-ISL system is assessed.

Our previous study in [6] is providing valuable contributions to the state of the art as follows:

- The 3D localization model applicable for the H-ISL system in the Cartesian coordinate system is derived;
- The H-ISL setup based on an L-shaped antenna array enables two kinds of range—coarse and fine—measurement and LoS estimation;
- A sensitivity analysis is conducted to evaluate the impact of ranging and phase measurement errors on LoS estimation.

This work extends our previous study presented in [6], offering significant contributions to the state of the art in the following ways:

- Initial work on peer-to-peer communication within the IOS domain, outlining the development scope of the L-shaped phased array;
- A network protocol stack is proposed to address key limitations of conventional ISL systems, including the following: (i) support for time division multiple access (TDMA) among more than two devices; (ii) integration of a metrology channel for range measurements; and (iii) network-wide time distribution;
- Coarse range measurements are performed via round-trip time (RTT) estimation in the baseband processor, following a calibration phase;
- Fine range measurements are achieved through phase-based techniques, utilizing frequency diversity for phase disambiguation;
- LoS estimation is enabled by combined ranging and phase difference measurements.

The main finding of our theoretical analysis is related to the sensitivity analysis of the LoS measurement to the inherent measurement errors. The sensitivity analysis allows design engineers to assess quantitatively the precision and accuracy of the measurement system in the presence of measurement errors.

This paper is structured as follows: First, the mathematical model for the 3D positioning system using an L-shaped antenna array is presented. Next, the H-ISL device and its corresponding network protocol are discussed, with a focus on the accuracy of ranging and phase measurements. A key research question explored is the extent to which measurement errors impact LoS precision and accuracy. To address this, LoS sensitivities to measurement errors are derived, followed by an interpretation of numerical results. Finally, conclusions are drawn, and directions for future work are outlined.

## 2. 3D Localization Models for L-Shaped Antenna Arrays

**Figure 1:**
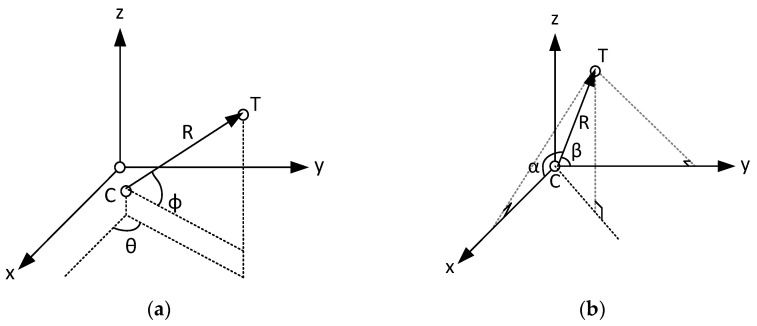
(**a**) The traditional model for 3D localization L-shaped array; (**b**) the 3D localization model proposed for standardization.

Figure 1a,b present a brief comparison between the traditional 3D localization model using an L-shaped array [20,21] and the model applicable to our H-ISL [22].

The reference 3D localization model in [20], set in aspherical coordinate system, consists of two satellites. One satellite is positioned at Cartesian coordinates *C* = (*x_C_*, *y_C_*, *z_C_*)—a first spacecraft that can be the servicer in the IOD scenario—and the other at *T* = (*x_T_*, *y_T_*, *z_T_*)—a second spacecraft that can be the serviced space device. The Cartesian coordinates of *C* and *T* can be retrieved from polar coordinate system as follows:

(1) $$\left[\begin{array}{c} x_{T}\\ y_{T}\\ z_{T} \end{array}\right]=\left[\begin{array}{c} cos\Phi cos\theta \\ cos\Phi sin\theta \\ -sin\Phi \end{array}\;\right]R+\left[\begin{array}{c} x_{C}\\ y_{C}\\ z_{C} \end{array}\right],$$

where *R* is the distance between the *C* and *T* satellites, *θ* is the azimuth of the incident signal, and *φ* is the angle of pitch. In general, the location of satellite *C*—provided by a Global Navigation Satellite System (GNSS)—can be considered as a known parameter. Still there are six unknowns to be found for 3D localization (1), which are *x_T_*, *y_T_*, *z_T_*, *R*, *φ*, and *θ*, respectively.

In our proposed 3D localization model, in Figure 1b, the origin of the Cartesian coordinate system is spacecraft *C*; thus the location of this satellite should be determined with other means, such as GNSS localization. The *T* spacecraft with the x-axis forms a plane; in this plane, one can measure the angle between the x-axis and the line-of-sight (here defined as the segment between point *C* and *T*). Let us denote this angle α, referred to as the LoS angle. Similarly, the y axis forms an angle with the LoS, this will be denoted by *β*. The resulting 3D localization model is as follows:

(2) $$\left[\begin{array}{c} x_{T}\\ y_{T}\\ z_{T} \end{array}\right]=\left[\begin{array}{c} \cos\alpha \\ \cos\beta \\ \sqrt{\sin^{2}\alpha +\cos^{2}\beta } \end{array}\right]R.$$

The derivation of the proposed 3D localization model in Equation (2) is described in Appendix A. In this model, to determine the position of spacecraft *T*, 3 measurements are necessary: the range measurement, *R*, and two LoS angles, α and *β*. In the next chapter, we present how these are measured by the H-ISL with an L-shaped antenna array.

## 3. The Hybrid-ISL Metrology Setup

**Figure 2:**
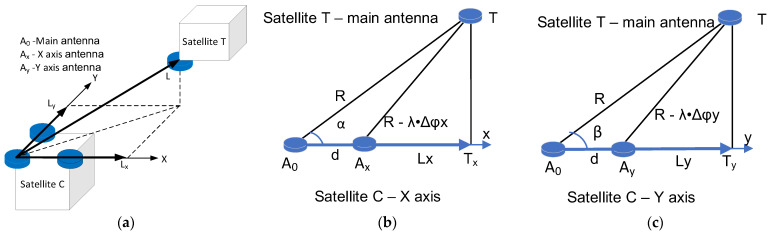
LoS measurement; (**a**) the projection of the LoS vector on the reference place; (**b**) the computation of the X-axis projection of the LoS; (**c**) the computation of the Y-axis projection of the LoS.

To compute the position of spacecraft *T* with respect to spacecraft *C*, the ISL system should provide radio frequency (RF)-based measurements: the range between the spacecraft, *R*, and the LoS angles on the L-shaped phase array, α and *β*. Refs. [22,23] give a suitable solution, where the ISL performs RF-based measurements by using 3 radio links between the two spacecrafts. There are three antennas mounted on spacecraft *C* and one antenna mounted on spacecraft *T*, as depicted in Figure 2a. The main antenna, *A_0_*, on both the *C* and *T* spacecrafts is used for range measurements, based on the round-trip time. The antennas mounted on spacecraft *C* constitute a reference plane. The location vector noted *L*, is projected onto the reference plane, furthermore the projection has two components along *x*- and *y*-axis *L_x_* and *L_y_* (see Figure 2b and Figure 2c, respectively). Note that the length of the segments are the coordinates of spacecraft *T* along the *x*- and *y*-axis. To compute the length of these two segments, two additional antennas, *A_x_* and *A_y_*, provide two phase-difference measurements, $\Delta \varphi_{x}$ and $\Delta \varphi_{y}$. The ISL’s module first computes the lengths *L_x_* and *L_y_* and then the LoS angles, α and *β*.

In Appendix B, the calculus for the projected lengths with respect to available measurements (range and phase difference) is given, yielding *L_x_* to be the following:

(3) $$L_{x}=\frac{R\lambda \Delta \varphi_{x}}{d}-\frac{\lambda^{2}\Delta \varphi_{x}^{2}}{2d}+\frac{d}{2},$$

where *λ* is the wavelength of the RF signal emitted by satellite *C*, *d* is the baseline distance (distance between the main antenna *A*_0_ and the *x*-axis antenna *A_x_*), *R* is the distance between the main *A*_0_ antennas, and Δ*φ_x_* is the phase difference measurement between *A*_0_ and *A_x_* on the carrier. From (3) the LoS angle along *x-*axis results, the following is derived:

(4) $$L_{x}=\frac{R\lambda \Delta \varphi_{x}}{d}-\frac{\lambda^{2}\Delta \varphi_{x}^{2}}{2d}+\frac{d}{2},$$

Similarly, the y-axis projection of the location vector *L* is as follows:

(5) $$\alpha =\arccos\left(\frac{L_{x}}{R}\right),$$

and, respectively, the counterpart LoS angle is as follows:

(6) $$\beta =\arccos\left(\frac{L_{y}}{R}\right).$$

## 4. A Hybrid-ISL System with Ranging and LoS Support

### 4.1. CCSDS’ Proximity-1 Network Stack

**Figure 3:**
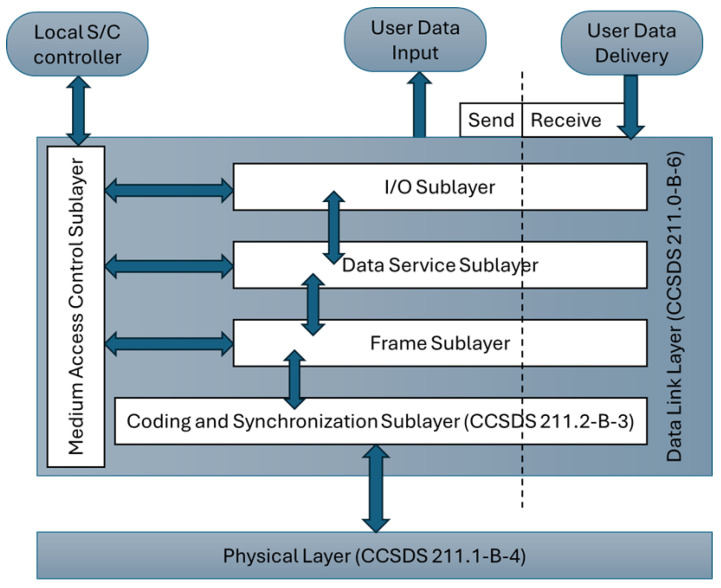
Simplified diagram of Proximity-1 layers.

Before presenting our recommendation for close-range (proximity) communications, we first provide a brief overview of the CCSDS Proximity communication suite to provide readers with relevant context for comparing the two protocol stacks. Additionally, we included a brief comparison between Proximity-1 and the proposed communication stack to offer the reader a quick overview of their differences. The Proximity-1 protocol stack, developed by the Consultative Committee for Space Data Systems (CCSDS), is intended to support reliable data exchange in space missions. The CCSDS has issued four recommendations pertaining to the Proximity-1 protocol [5,6,7,8]. The Proximity-1 protocol stack consists of two communication layers, as illustrated in Figure 3, adapted from [6].

- Physical Layer (PHY): at the lowest level, the PHY defines the hardware interfaces and signaling characteristics for transmitting data over space communication links. It specifies parameters such as modulation, coding, and frequency allocation to ensure reliable transmission in the harsh space environment.
- Data Link Layer (DLL): it provides mechanisms for framing, error control, and flow control to ensure the integrity and efficiency of data transmission. It ensures reliable delivery of data using Automatic Repeat reQuest (ARQ) and provides services for both acknowledged and unacknowledged data transfers. It includes protocols for packetization, error detection and correction, and management of data link connections. This layer furthermore consists of a Medium Access Control (MAC) and Coding and Synchronization sublayers.

Let us provide a comparison between CCSDS’s Proximity-1 suite and our proposed hybrid-ISL system protocol stack in advance of presenting the letter. The comparison is summarized in Table 1.

### 4.2. Proposed Network Protocol Stack for Hybrid-ISL System

**Figure 4:**
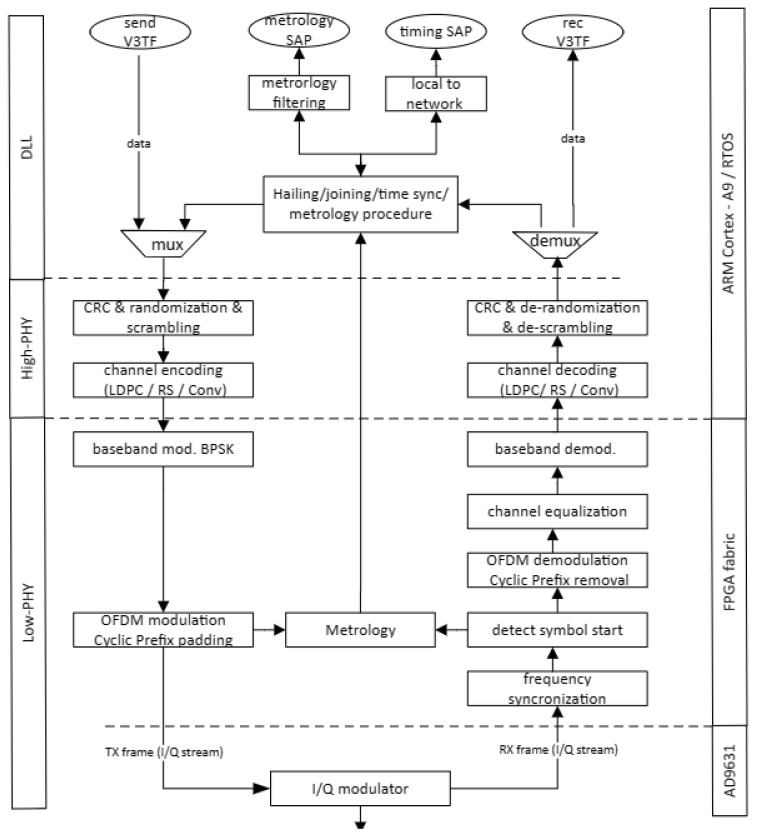
Network protocol stack and its hardware partitioning.

In Figure 4, the H-ISL’s network protocol stack is depicted, highlighting the layers on the left side and the corresponding hardware partitioning on the right side, implemented on a software defined radio (SDR) platform consisting of a ZCU102 FPGA development board [24] and the AD-FMCOMM-S5 daughter board [25] featuring the AD9361 highly integrated RF transceiver chip. The protocol stack consists of the PHY and DLL layers, where the PHY is segmented into the High-PHY and Low-PHY sublayers.

The use of a DLL layer is indicated to enable data communication, metrology, and timing (for accurate time stamping) channels. Each channel can be accessed by its service access points (SAPs) (i.e., Metrology SAP and Timing SAP). The DLL is also responsible for hailing, joining, and time synchronization functionalities in the network.

The data flow in the PHY layer is as follows. On the transmit side of the PHY (downlink (DL) from the viewpoint of a base station/access point):

- The stream from the DLL will be first randomized and then scrambled;
- After that channel encoding is performed, possible candidates for channel encoding are the Reed–Solomon (RS), low-density parity check (LDPC), or convolutional coding, in compliance with [5];
- The channel encoding is followed by a Binary Phase Shift Keying (BPSK) baseband modulation;
- The 1024 subcarrier data is then applied to a 1024-point Inverse Fast Fourier Transform (IFFT) block, implementing the orthogonal frequency division modulation (OFDM). Note: the sub-carrier spacing is 15 kHz and a 15.36 MHz sampling frequency and 9 MHz channel width is applied. The first 212 and, respectively, the last 211 subcarriers are used as a guard interval, and the remaining 600 subcarriers are used for data transmission. The direct current (DC) component is not modulated. Each OFDM symbol is preceded by a cyclic prefix (CP), with a length of 72 samples (4.68 µs); thus the total length of a symbol is 1096 samples (with a total duration of 71.35 µs);
- The resulting symbol is then modulated into RF frequencies by the AD9631 transceiver chip.

On the receiver side of the PHY (up-link (UL) from the viewpoint of a base station/access point):

- Carrier frequency synchronization is is carried out as in [26]
- The beginning of a symbol in the I/Q stream is detected by an autocorrelation function of the prescribed and the received preamble;
- The cyclic prefix is removed;
- The OFDM demodulation takes place in a 1024-point FFT block;
- Channel equalization is carried out based on the received preamble;
- Baseband demodulation;
- Channel decoding;
- Descrambling and normalization is executed.

### 4.3. PHY Support for Coarse and Fine Ranging

#### 4.3.1. Coarse Range Measurement

**Figure 5:**
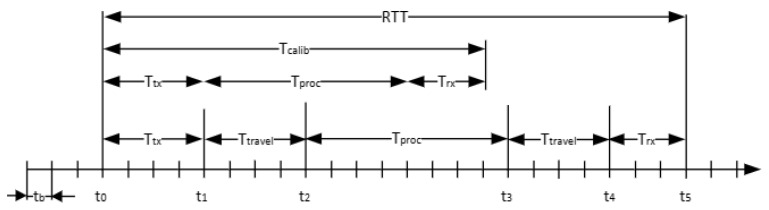
Coarse range measurement timing diagram.

The coarse range estimation is based on the round-trip time (RTT) measurement [27] implemented in the PHY layer. This measurement provides the initial estimation of the distance between the 2 satellites (i.e., distance between satellite *C* and *T*). The RTT measurement and the computation of the effective travel time is illustrated in Figure 5.

The baseband processor is clocked at F_B_, which corresponds to a time base resolution of t_b_. Noticeable events in the timing diagram are as follows:

- t_0_—the baseband processor sent the OFDM burst to the analog front-end;
- t_1_—the OFDM burst is aired on the antenna;
- t_2_—the burst is received by the counterpart PHY;
- t_3_—the counterpart PHY processes the OFDM burst and airs a response;
- t_4_—the front-end receives the OFDM burst;
- t_5_—the PHY detects the beginning of the OFDM burst.

The time required by the analog front-end to send and receive an OFDM burst, noted *T_tx_* and *T_rx_* in the figure, are deterministic. Also, the time required by the counterpart PHY, *T_proc_*, to process and prepare an OFDM burst in response is deterministic. With a previous calibration, these deterministic values can be obtained, *T_calib_*~*T_tx_* + *T_proc_* + *T_rx_*. The PHY starts and stops a counter that is clocked at the baseband processors frequency, at t_0_ and t_5_, respectively, to measure the RTT. The effective distance can be estimated from the travel time, *T_travel_*, that is the difference between the RTT and the previously determined calibration time, knowing the propagation speed of radio waves in vacuum to be c~300,000,000 m/s:

(7) $$R_{coarse}=T_{travel}\cdot c=\frac{\left(RTT-T_{calib}\;\right)}{2}\cdot c$$

The accuracy of the RTT measurement is given by the smallest time interval the baseband can provide. The H-ISL implementation uses a sample frequency of *F_s_* = 61.44 MHz, which is also the operating frequency of the baseband processor. Thus, the achievable resolution one may obtain is approximately 2.44 m. 

#### 4.3.2. Fine Range Measurement

**Figure 6:**
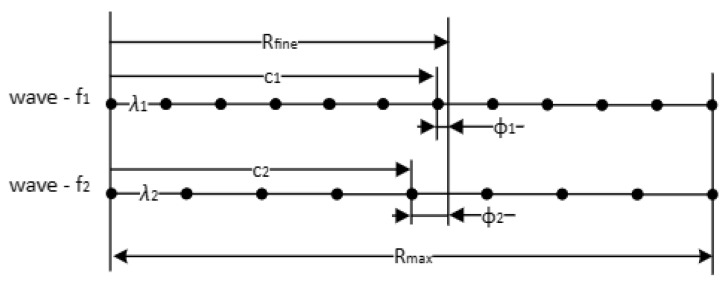
Fine range measurement using frequency diversity (for phase disambiguation).

Fine range estimation, presented in Figure 6, is based on frequency diversity for phase disambiguation. Two radio waves with different wavelengths are used, which are noted wave—*f*_1_ and wave—*f*_2_ in the picture. The least common multiple of the two wavelengths gives the maximum unambiguous fine measurement range, *R_max_*. Let us consider that the actual distance to be measured is *R_fine_*. This distance shall result in a frequency difference on a main and a secondary antenna, in *φ*_1_ and *φ*_2_ on wave—*f*_1_ and on wave —*f*_2_, respectively. The full cycles *n*_1_ and *n*_2_ on wave—*f*_1_ and wave—*f*_2_, respectively, can be computed by solving a simple equation, which yields the measurements:

(8) $$R_{fine}=\lambda_{1}\cdot n_{1}+\varphi_{1}\frac{\lambda_{1}}{2\pi }=\lambda_{2}\cdot n_{2}+\varphi_{2}\frac{\lambda_{2}}{2\pi }$$

One can observe that in Equation (8) there are 2 unknowns, namely the full cycles *n*_1_ and *n*_2_, and just one equation. There are several approaches to obtain the solution, i.e., what is given in [23]. The solution in [23] discards that Equation (8) can be further constrained; the values of *n*_1_ and *n*_2_ are natural integer values. Thus, a simple search algorithm presented in Algorithm 1 suffices for a solution. A numerical example is given in Appendix C.

By using frequency diversity to solve phase disambiguation, virtually a new wavelength is generated that is as follows [28]:

(9) $$\lambda_{v}=\left|\frac{\lambda_{1}\lambda_{2}}{\lambda_{1}-\lambda_{2}}\right|,$$

while the distance (maximum range) covered without ambiguity is extended to the following:

(10) $$R_{max}=\frac{2}{\left|\frac{1}{\lambda_{1}}-\frac{1}{\lambda_{2}}\right|}.$$

Let us consider two carrier frequencies *f*_1_ = 2050 MHz and *f*_2_ = 2150 MHz. Using these two frequencies, the virtual wavelength results in approximately 6.8 mm, while the maximum range that can be measured without ambiguity is R_max_ is 6 m.
**Algorithm 1** Search algorithm for solving full cycles in Equation (8)**Inputs: **$\varphi_{1}$$\;\mathrm{and}\;\varphi_{2}$—phase measurements; N—maximum number of cycles; ε—threshold     **for** n_2_ = 0:N           $n_{1}=(\lambda_{2}$$*\;n_{2}+{(\varphi }_{1}$$-\varphi_{2})/2\pi )$$/\lambda_{1}$;          **if** (**abs**(n_1_− **round**(n_1_)) < ε)               **break**;         **end if;**      **end for;** **return** n_1_ and n_2_

#### 4.3.3. Phase-Difference Measurement

Let us recall the metrology setup illustrated in Figure 2b,c. To compute or estimate the LoS angles, α and β, two additional phase-difference measurements are required—one along the *x*-axis and the other along the *y*-axis—resulting in phase differences $\Delta \varphi_{x}$ and $\Delta \varphi_{y}$, respectively. The signal transmitted from satellite *T* is received by the main antenna *A*_0_ on satellite *C*, as well as by two additional antennas positioned along the x- and y-axes, *A_x_* and *A_y_*, with corresponding phase differences of $\Delta \varphi_{x}$ and $\Delta \varphi_{y}$. The phase difference measurement is carried out by detecting the zero crossing of a carrier received by antenna *A*_0_ and its delayed version on antenna *A_x_* or *A_y_* and measuring the time difference between two consecutive zero crossings. With this procedure the achievable phase resolution *R_φ_* is inversely proportional to the frequency of the baseband sampling frequency, in our setup *F_s_* = 15.36 MHz, and proportional to the carrier frequency, *F_C_*. Expressed in rad/s, the resolution yields the following:

(11) $$R_{\varphi }=2\pi \frac{F_{C}}{F_{S}}.$$

For example, with a carrier frequency *Fc* = 25 kHz, approximately a 0.01 rad/s phase difference resolution may be achieved. Note, that fine range measurement may be carried out based on the received OFDM symbol, just as in [29].

## 5. Sensitivity Analysis with Respect to Measurement Errors

### 5.1. Sensitivity of the LoS Angle with Respect to Range Measurement Errors

As shown in the previous section, the LoS angles are computed from three measurements: range, *R*, and the phase difference between the main and secondary antenna placed on x- and y-axis, Δ*φ_x_* and Δ*φ_y_*, respectively. The baseline distance, *d*, and the wavelength, *λ*, are considered parameters for the sensitivity analysis.

By definition, the sensitivity of the LoS angle α (or its counterpart on the y-axis, *β*) with respect to the range *R* is as follows:

(12) $$S_{R}^{\alpha }=\frac{R}{\alpha }\frac{d\alpha }{dR}=\frac{R}{\alpha }\arccos^{\prime }\left(\frac{\lambda \Delta \varphi }{d}-\frac{\lambda^{2}\Delta \varphi^{2}}{2dR}+\frac{d}{2R}\right).$$

The impact of the range measurement error, ε_R_, on the LoS angle error, ε_α_, is as follows:

(13) $$\frac{\varepsilon_{\alpha }}{\alpha }S_{R}^{\alpha }=\frac{\varepsilon_{R}}{R}.$$

**Figure 7:**
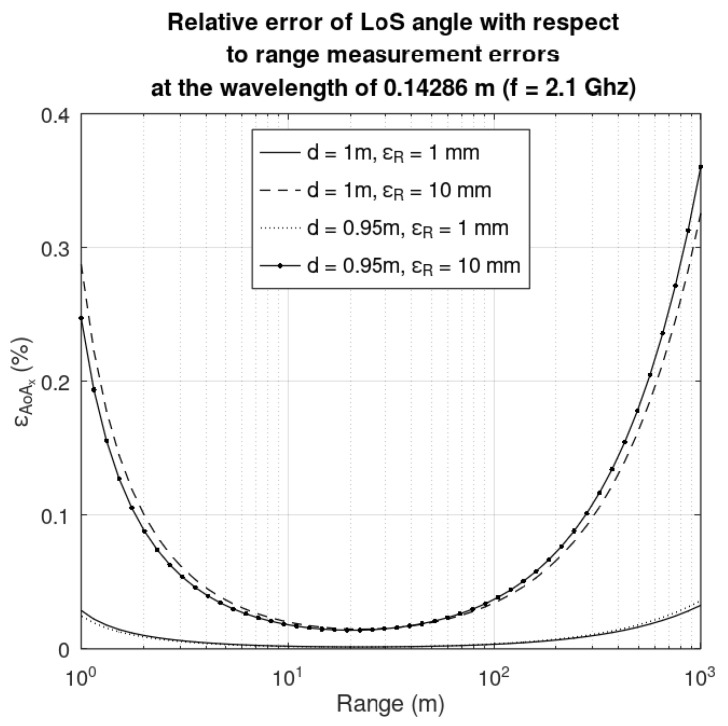
Relative error of the LoS angle with respect to a baseline range of d = 0.95 m and 1 m, respectively, and an absolute measurement error ε*_R_* of 1 mm and 10 mm, respectively.

As seen in the Equation (13), a range measurement error will inherently result in an error in the LoS angle. Sources of ranging errors are twofold: one can be the misplacement of the antenna (on the face of a satellite many instruments and other antennas must be mounted, so in the case of antenna placement constraints, the antenna may be placed closer as indicated in an initial design); second is the resolution of the fine ranging. In Figure 7 the LoS angle relative error with respect to the range is depicted for the next situations: the baseline is *d* = 0.95 m and 1 m, respectively, and the absolute measurement error ε*_R_* is 1 cm and 10 cm, respectively. A shorter baseline will result in smaller LoS angle errors for small ranges; for large ranges the LoS angle errors are negligible.

### 5.2. Sensitivity of the LoS Angle with Respect to Phase Measurement Errors

By definition, the sensitivity of the LoS angles, with respect to the baseline *R*, is as follows:

(14) $$S_{\Delta \varphi }^{\alpha }=\frac{\Delta \varphi }{\alpha }\frac{d\alpha }{d\Delta \varphi }=\frac{\Delta \varphi }{\alpha }\arccos^{\prime }\left(\frac{\lambda \Delta \varphi }{d}-\frac{\lambda^{2}\Delta \varphi^{2}}{2dR}+\frac{d}{2R}\right).$$

The impact of the range measurement error on the LoS angle is expressed as follows:

(15) $$\frac{\varepsilon_{\alpha }}{\alpha }S_{\Delta \varphi }^{\alpha }=\frac{\varepsilon_{\Delta \varphi }}{\Delta \varphi }$$

**Figure 8:**
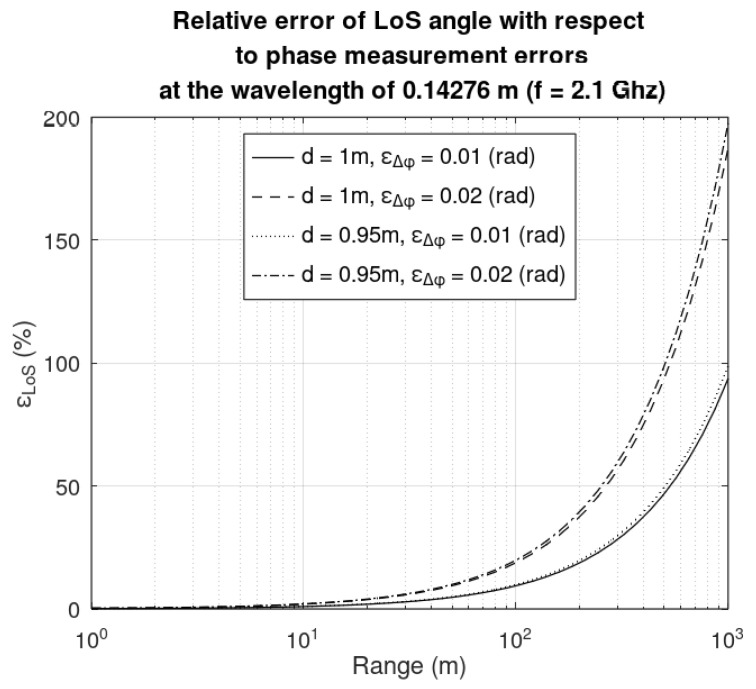
Relative error of the LoS angle with respect to a baseline range of d = 0.95 m and 1 m, respectively, and phase difference measurement errors of 0.01 rad and 0.02 rad, respectively.

As seen in Equation (15), a phase difference measurement error will inherently result in an error in the LoS angle. Sources of phase difference measurement may be caused by the antenna placement (due to restrictions, the antennas are not placed at the indicated distance of 1 m) and the phase difference measurements resolution (in this case a 0.01 rad/s resolution was suggested). In Figure 8 the relative error of the LoS angle with respect to the range is depicted for the next situations: the baseline is *d* = 0.95 m and 1 m, respectively, and absolute measurement error of the phase difference is pi/20 rad and pi/50 rad, respectively. A shorter baseline will result in smaller LoS angle errors for large phase differences; for small phase differences the LoS angle errors are negligible.

## 6. Conclusions

The variation in the antenna baseline has an impact on the accuracy of the LoS angle and subsequently in the 3D positioning, with respect to both range and phase difference measurement errors. In the case of the range measurement errors, the effect on LoS angle accuracy is noticeable especially at small distances under 1 m and remains practically constant (in percentage) once the distances increase over 10 m. A smaller baseline has a positive effect on the LoS angle determination for small distances. In the case of phase measurement errors, the effect on LoS angle accuracy is noticeable especially at large distances over 100 m and remains practically constant (in percentage) once the distances are less than 10 m. A smaller baseline has a negative effect on the LoS angle determination for large distances. As further work, we plan to develop an experimental setup on a software-defined radio platform that may host the proposed network protocol stack to validate our theoretical findings. The results should be corroborated through modelling and tests.

## Figures and Tables

**Table 1:** Comparison of the Proximity-1 protocol and the proposed Hybrid-ISL.

		Proximity-1	Hybrid-ISL
PHY layer	Frequency band	UHF	S-band
	Bandwidth	1.5 MHz	10 MHz
	Data rate	up to 1 MBps	>1 MBps
	Modulation scheme	OFDM	PSK
	Ranging support	yes	yes
	Line-of-sight support	no	yes
DLL layer	Synchronization	Standardized	Custom algorithm
	Channel access	Combined time/frequency division	Frequency division
	Channel coding	Convolutional	Read–Solomon (configurable)
	Quality of service	ARQ/expedited	expedited

**Figure A1:**
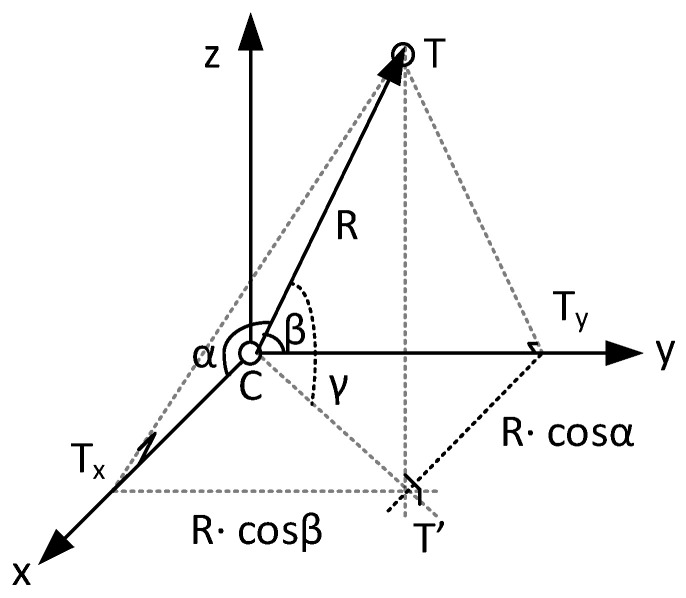
3D positioning using range and LoS angles.

**Figure A2:**
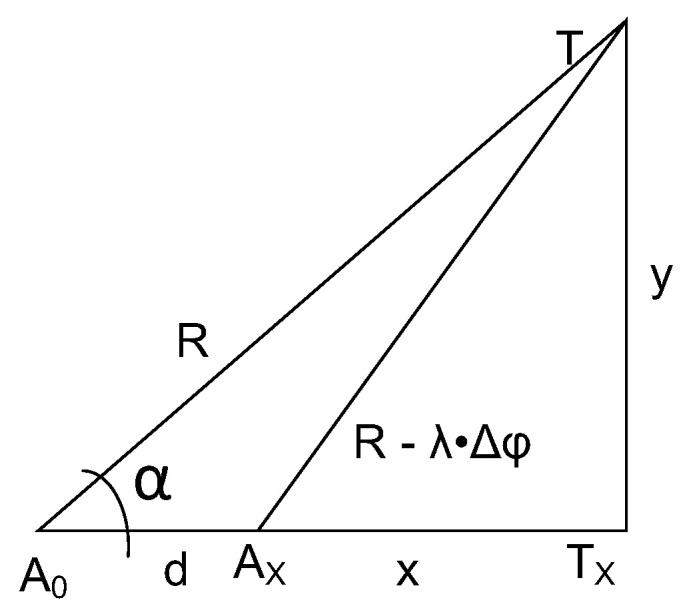
Computation of the projection lengths from range and phase difference measurements.

## Data Availability

This is a theoretical study, no data was generated or used.

## Appendix A. The Derivation of the Proposed 3D Localization Model

Let us consider the 3D positioning model in Figure A1. The origin of the Cartesian coordinate system is one of the spacecrafts equipped with a hybrid-ISL that can measure the range *R* and compute the angle from phase difference measurements.

The coordinates of the *T* spacecraft along the *x-* and *y-*axis are straightforward to compute. One can consider the projections of point *T_on_ x-* and *y-*axes to be *T_x_* and *T_y_*, respectively. The right-angle triangles *CTT_x_* and *CTT_y_* have known angles α and *β* and a known hypotenuse equal to the range *R*. Thus, the segments *CT_x_* and *CT_y_*, that are the coordinates on the *x*-, *y*-axis, respectively, can be computed as follows:

(A1) $${CT}_{x}=R\cdot cos\alpha ;{CT}_{y}=R\cdot cos\beta .$$

Let us consider the projection *T′* of point *T* on the reference plain defined by *x-* and *y-*axes. The length of diagonal *CT′* of rectangle *CT_x_T′T_y_* is as follows:

(A2) $${CT}^{\prime }=R\cdot \sqrt{{cos}^{2}\alpha +{cos}^{2}\beta }.$$

Applying Pythagoras theorem on the triangle *CTT′* with a length of *TT′* that is equal to the *z-*axis coordinate yields the following:

(A3) $$TT\prime =R\cdot \sqrt{{sin}^{2}\alpha +{cos}^{2}\beta }.$$

## Appendix B. The Calculus for the Projection Lengths

Let us consider the *A_0_TT_x_* and *A_x_TT_x_* right-angle triangles in Figure A2. Let us note the length of segments *A_0_T_x_*, *TT_x_*, and *A_0_A_x_* by *x*, *y*, and *d*, respectively.

The length of the hypotenuse, *R*, is known from round-trip time measurements. A phase difference measurement, Δ*ϕ,* of a signal transmitted from *T* and arriving at points *A*_0_ and *A_x_* is carried out. The length of segment *TA_x_* is less than the length of segment *TA*_0_ with the product of the phase difference Δ*ϕ* and the wavelength *λ*.

Applying Pythagoras theorem on the triangles *A_0_TT_x_* and *A_x_TT_x_* one obtains the following equation system:

(A4) x2+y2=R2x−d2+y2=R−λΔϕ  2.

Expanding the second equation one can get the following:

(A5) $$x^{2}-2xd+d^{2}+y^{2}=R^{2}-2R\lambda \Delta \phi +\lambda^{2}\Delta \phi^{2}.$$

On can observe that *x*^2^ + *y*^2^ on the left side is equal to *R*^2^ on the right side; thus, this can be optimized and Equation (15) yields the following:

(A6) $$-2xd=-2R\lambda \Delta \phi +\lambda^{2}\Delta \phi^{2}-d^{2}.$$

Finally, the length of the projection on the *x-*axis is as follows:

(A7) $$x=\frac{R\lambda \Delta \phi }{d}-\frac{\lambda^{2}\Delta \phi^{2}}{2d}+\frac{d}{2}$$

## Appendix C. Fine Range Measurement—A Numerical Example

Let us consider the two carrier frequencies *f*_1_ = 2050 MHz and *f*_2_ = 2250 MHz applied for frequency diversity. Note that these S-band frequencies are in the frequency range recommended by the Space Frequency Coordination Group for in situ lunar links [30]; thus most likely these frequencies may be used for proximity communications. The resulting wavelengths *λ*_1_ and *λ*_2_ are equal to 145.85 mm and 132.89 mm, respectively. The resulting virtual wavelength according to Equation (9) is *λ_virtual_* = 12.93 mm, while the maximum range, *R_max_*, is approximately 3 m according to Equation (10). Let us consider the actual distance *D* to be 2 m. For this distance, wavelength *λ*_1_ and *λ*_2_ will have *k*_1_ = 13 and *k*_2_ = 14, respectively, full cycles—these are the unknown to be determined, while the remaining phases are approximately 0.61 and 0 rad/s (these are the input for the disambiguation algorithm). Runing the algorithm in Algorithm 1 will return the full cycles on the two wavelengths. Knowing the number of cycles and the remaining phase, the fine measurement procedure returns the estimated distance *d*. This numerical example is summarized in Table A1.

Let us consider a second scenario, where the carrier frequencies are *f*_1_ = 2050 MHz and *f*_2_ = 2150 MHz, and the distance in question is 1.56 m. In this case the virtual wavelength results in 6.8 m, while the maximum range is about 6 m. There are 10 and 11 full cycles, respectively, on the two waves, and the phase measurements are 0.6 and 0.15 rad/s. The disambiguation algorithm returns 10 and 11 cycles, just as expected, while the estimated distances on the carriers are 1.48 m and 1.56 m, respectively. This numerical example is also summarized in Table A1.

**Table A1:** Numerical examples to illustrate disambiguation process for fine ranging.

	Example 1		Example 2		Formula
	Carrier 1	Carrier 2	Carrier 1	Carrier 2	
phase meas. resolution, R_φ_	0.01 rad/s				
actual distance, D	2 m		1.56 m		
frequency (GHz), f_1_ and f_2_	2.05	2.25	2.05	2.15	
wavelength (mm), λ_1_ and λ_2_	145.85	132.89	145.85	139.89	λ_n_ = c/f_n_
virtual wavelength, λ_virtual_ (mm)	12.93		6.8		λ_virtual_ = |λ_1_ − λ_2_|
maximum range (m), R_max_	3		6		R_max_ = 2/(1/λ_1_ − 1/λ_2_)
full cycles, k_1_ and k_2_	13	14	10	11	k = floor(D/λ))
remaining phase (rad/s), φ_r1_ and φ_r2_	0.6	0	0.6	0.15	φ_rn_ = 2π(D mod λ_nn_)
*input to algorithm*	60	0	60	15	N = floor(φ_r_/R_φ_)
*output of the algorithm*	13	14	10	11	
estimated distance (m), d_1_ and d_2_	1.92	2	1.48	1.54	d = λ_n_k_n_ + φ_rn_ R_φ_ λ_n_/2/π
relative error (%)	4	0	5	1	E = |D − d_n_|/D
